# Unusual cause of filling defect in pulmonary artery: pulmonary artery sarcoma

**DOI:** 10.11604/pamj.2020.35.41.19563

**Published:** 2020-02-12

**Authors:** Cisel Yazgan, Hakan Ertürk, Aysenaz Taskin

**Affiliations:** 1 Department of Radiology, Faculty of Medicine, Hacettepe University, Ankara, Turkey; 2 Department of Radiology, Faculty of Medicine, Kastamonu University, Kastamonu, Turkey; 3 Department of Radiology, Atatürk Chest Diseases and Thoracic Surgery Training and Research Hospital, 06280 Ankara, Turkey; 4 Department of Chest Diseases, Atatürk Chest Diseases and Thoracic Surgery Training and Research Hospital, 06280 Ankara, Turkey

**Keywords:** Pulmonary artery, intimal sarcoma, pulmonary thromboembolism

## Abstract

Pulmonary artery (PA) sarcoma is an extremely rare malignant tumor of pulmonary artery. It is often misdiagnosed as pulmonary thromboembolism (PTE) because of its clinical and imaging features which are quite similar to PTE. Multimodality diagnostic imaging and recognition of specific imaging characteristics with appropriate clinical suspicion are required to make correct diagnosis. In this report, we present a case of PA sarcoma with imaging and clinical features as well as emphasize significance of using multimodality imaging.

## Introduction

Pulmonary artery (PA) sarcoma is a rare tumor originating from mesenchymal cells of pulmonary artery. Patients with PA sarcoma usually present with nonspecific pulmonary symptoms such as dyspnea, cough and haemoptysis. In addition, main manifestation of PA sarcoma on computed tomography pulmonary angiography (CTPA) is filling defect, which mimics PTE. Due to its nonspecific clinical and imaging features, early diagnosis is tough preoperatively [1]. Delays in the diagnosis cause advanced stage presentation of tumor and unnecessary anticoagulation therapy. Therefore, it is crucial to differentiate PA sarcoma from PTE based on imaging and clinical features. Herein we reported a case of PA sarcoma with characteristic CTPA features and emphasize differential diagnosis.

## Patient and observation

**Figure 1:**
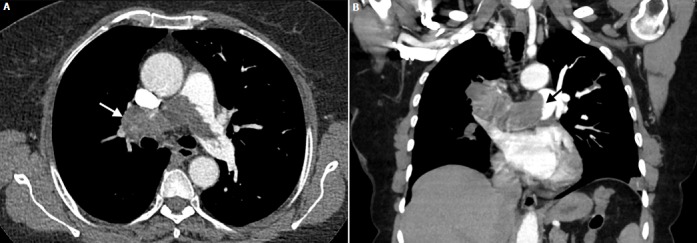
Axial (A) and coronal (B) CT angiography images: A) a large low density filling defect occupying lumen of the pulmonary trunk, right and left pulmonary arteries with expansion of right pulmonary artery (white arrow); B) protrusion of the proximal end of the lesion is also observed (black arrow)

**Figure 2:**
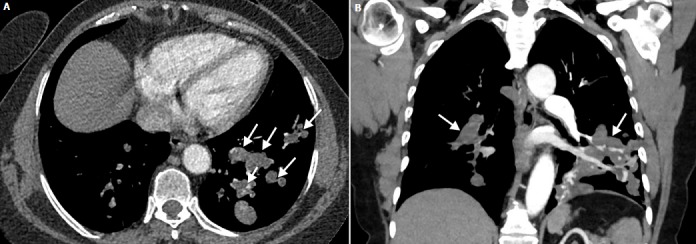
Extraluminal extension of the tumor (A) and grape like shape of involved segmental and subsegmental branches of PA (white arrows) are observed on axial (A) and coronal reconstructed (B) CT images

A 64-year-old woman admitted to our hospital with a 1 month history of dyspnea. Her medical history also revealed that she received antibiotic and anticoagulant therapy for diagnosis of PTE and pneumonia in the past 2 weeks but had no clinical improvement. Physical examination was unremarkable. Laboratory test showed elevated D-dimer level (1990 ng/ml) and C-reactive protein (16.6 mg/dl). Levels of tumor markers were normal. Transthoracic echocardiography showed high pulmonary artery pressure (65 mmHg). She underwent CTPA with suspicion of pulmonary emboli. Thrombus at the lower extremities was not detected on venous Doppler ultrasound. CTPA demonstrated intraluminal filling defect at the pulmonary trunk extending right and left pulmonary arteries. Expansion of the involved pulmonary arteries and extension to segmental-subsegmental branches were also noted (Figure 1, Figure 2). In addition, increased fluorodeoxyglucose (FDG) uptake was detected at the level of the filling defects by subsequent positron emission tomography (PET) - computed tomography (CT) scan. The patient underwent tru-cut lung biopsy and histopathologic examination revealed pulmonary artery sarcoma. Chemotherapy was started but she died two months after initial presentation.

## Discussion

Pulmonary artery (PA) sarcoma arises from the intimal layer of pulmonary trunk and main pulmonary arteries. It has very poor prognosis and only chance of survival is early diagnosis with radical surgical resection. However, clinical manifestations of PA sarcoma are nonspecific and imaging features are very similar to those of PTE. In a study, based on imaging and clinical criteria, 47% of the patients were initially considered to have PE including 39% receiving anticoagulant therapy [1]. CTPA is widely used in the assessment of patients with dyspnea and suspected PTE. Both PTE and PA sarcoma cause intraluminal filling defect on CTPA. Although CTPA features of PA sarcoma are similar to PTE with respect to intraluminal filling defects, some subtle differences can raise suspicion of PA sarcoma. Expansion of involved artery and protrusion of proximal end of mass are characteristic imaging features suggestive of PA sarcoma [2]. In addition, presence of extraluminal tumor extension supports a diagnosis of PA sarcoma [3]. In contrast to PTE, PA sarcoma usually show unilateral and central presentation with higher attenuation filling defect. When tumor extends to peripheral branches, it leads to pseudoaneurysm and grape like appearance on CT images [4]. This sign has been reported by Liu *et al.* as 100% specific to PA sarcoma on magnetic resonance imaging (MRI). Moreover, the authors believed that this feature shows malignant nature of sarcoma, which grows rapidly and invades to segmental-subsegmental branches of PA [5]. In the presented case, pseudoaneurysm and grape like appearance of involved peripheral PA were demonstrated by coronal reformatted images. In spite of these characteristic CT features, differentiating PA sarcoma from PTE only by CTPA would be difficult. Some authors have reported that PET-CT may be a useful modality in differentiating PA sarcoma from PTE [6]. Compared with PTE, tumor has higher metabolic activity and demonstrates increased F-FDG uptake as in our case. PET-CT also has the advantage of identifying the presence of other foci and distant metastasis. In addition, MRI is a highly specific imaging tool in diagnosis of PA sarcoma. Enhancement features of tumor on T1-weighted images with gadolinium can help to distinguish PA sarcoma from PTE [7]. However, it requires a longer breath hold time which can be a problem for the patients who are heavily dyspneic. In addition to imaging characteristics clinically, its insidious onset, increased serum inflammatory marker levels, lack of predisposing factors and inadequate response to thrombolytic therapy may help to differentiate PA sarcoma than PTE.

## Conclusion

In spite of its less prevalence, PA sarcoma should be considered in differential diagnosis of the patients presenting with PTE particularly in those with inadequate response to thrombolytic therapy. Combining various imaging modalities and recognizing of characteristic CTPA features associated with high suspicion are important in precise diagnosis of PA sarcoma and preventing delays.
